# Cellulose Nanofibril/Carbon Nanomaterial Hybrid Aerogels for Adsorption Removal of Cationic and Anionic Organic Dyes

**DOI:** 10.3390/nano10010169

**Published:** 2020-01-19

**Authors:** Zhencheng Yu, Chuanshuang Hu, Anthony B. Dichiara, Weihui Jiang, Jin Gu

**Affiliations:** 1 College of Materials and Energy, South China Agricultural University, Guangzhou 510642, China; 2 School of Environmental and Forest Sciences, University of Washington, Seattle, WA 98195, USA

**Keywords:** cellulose nanofibrils, graphene nanoplates, carbon nanotubes, aerogel, organic dyes, adsorption

## Abstract

Advances in nanoscale science and engineering are providing new opportunities to develop promising adsorbents for environmental remediation. Here, hybrid aerogels are assembled from cellulose nanofibrils (CNFs) and carbon nanomaterials to remove cationic dye methylene blue (MB) and anionic dye Congo red (CR) in single and binary systems. Two classes of carbon nanomaterials, carbon nanotubes (CNTs) and graphene nanoplates (GnPs), are incorporated into CNFs with various amounts, respectively. The adsorption, mechanics and structure properties of the hybrid aerogels are investigated and compared among different combinations. The results demonstrate CNF–GnP 3:1 hybrid exhibits the best performance among all composites. Regarding a single dye system, both dye adsorptions follow a pseudo-second-order adsorption kinetic and monolayer Langmuir adsorption isotherm. The maximal adsorption capacities of CNF–GnP aerogels for MB and CR are 1178.5 mg g^−1^ and 585.3 mg g^−1^, respectively. CNF–GnP hybrid show a superior binary dye adsorption capacity than pristine CNF or GnP. Furthermore, nearly 80% of MB or CR can be desorbed from CNF–GNP using ethanol as the desorption agent, indicating the reusability of this hybrid material. Hence, the CNF–GnP aerogels show great promise as adsorption materials for wastewater treatment.

## 1. Introduction

Dyes are colored organic chemicals typically classified as anionic (acid, reactive, and direct dyes), cationic (all basic dyes), and non-ionic (dispersed dyes) based on their charge upon dissolution in aqueous solutions [1]. These complex molecules are widely used in many industrial fields, such as textile, paper, leather tanning, food processing, plastics, cosmetics, rubber, and printing. The contamination of the hydrosphere with dyes raises serious environmental and sanitary concerns due to their ubiquity, toxicity and deleterious effects on photosynthetic activity in aquatic life due to decreased sunlight penetration [2]. Particularly, methylene blue (MB), one of the most widely used basic dyes in the printing and textile industries, can cause a variety of harmful effects, such as eye burns, gastrointestinal tract and skin irritation [3]. As a typical direct azo dye, Congo red (CR) is mainly applied in a relatively large dosage for dyeing biological samples, and can increase the risk of cancer if absorbed into the human body [4]. Currently, there are various industrial methods for treating waste dye solutions, including adsorption, microbial treatment, chemical oxidation or reduction, flocculation precipitation, ozone oxidization, chemical precipitation, nanofiltration, catalytic degradation etcetera [5,6,7]. Due to its simple operation and low cost, adsorption is widely used as a commercial way to remove organic dyes from aqueous solutions [8].

Recently, carbon nanomaterials have attracted a broad interest in pollutant adsorption. Among them, carbon nanotubes (CNTs) are comprised of one or several sheets of hexagonally packed carbon atoms rolled into concentric seamless cylinders; these exhibit strong mechanical properties, a high aspect ratio, excellent chemical stability and a large specific surface area, which make them desirable for dye adsorption [9,10,11]. The adsorption mechanism of organic compounds on carbon nanomaterials can be described as an interplay between different intermolecular forces (i.e., hydrophobic, van der Waals forces, π–π bonding, hydrogen bonding, and electrostatic interactions), whose contributions depend on the adsorbate nature and the surface chemistry of the nano-adsorbent [12]. Another type of carbon nanomaterial, graphene, is a planar honeycomb-shaped nanomaterial consisting of six-member rings with a large specific surface area (theoretical value of 2630 m^2^ g^−1^) [13]. It is more easily prepared than CNT and shows an extensive application prospect in the removal of organic and inorganic pollutants [14] and dyes [15,16]. Graphene could be modified to adsorb pollutants via π–π, electrostatic interaction and hydrogen bonding. However, the cost of a carbon-based nano-adsorbent is high and the regeneration treatment of spent materials is often challenging [17]. Due to strong π–π bonds and van der Waals forces, graphene sheets tend to condense and restack. CNTs often group into bundles and tangle due to the same reason. Therefore, the available specific surface area of these carbon nanomaterial is much lower than the theoretical value, greatly restricting their practical applications [18]. Thus, providing a recycling function and reducing the aggregation potential of the carbon nanomaterials are vital ways to design high-efficiency adsorbents.

Nanocelluloses are a class of renewable nanomaterials derived from the most abundant natural polymer on earth. Their high specific surface area, bioavailability and surface reactivity make nanocelluloses interesting materials for pollutant adsorbents [19]. Since unmodified nanocelluloses expose abundant surface hydroxyl groups and perhaps other negatively charged groups such as sulfate or carboxylate groups depending on the preparation procedure [20,21], they are well-suited for the adsorption of cationic molecules. Recently, high surface-area cellulose nanofibril (CNF) aerogels prepared via 2,2,6,6-tetramethylpyperidine-1-oxyl (TEMPO) oxidation were shown to absorb cationic malachite green (MG) dye (212.7 mg g^−1^) due to electrostatic interactions between MG and negatively charged oxygen moieties on the CNF surface [22]. To make nanocellulose adsorbents for anionic dye, chemical modifications, such as introduction of positively charged amino groups, are usually required [23]. However, these positively charged nanocelluloses are no longer suitable for cationic dye adsorption.

While the above studies demonstrate the individual efficacy of carbon nanomaterials and nanocellulose adsorbents, improved adsorption performance and cost optimization may be realized by combining these nanomaterials together. Since carbon nanomaterials and nanocellulose exhibit different affinities for given molecules; hence, their combination can increase the variety of pollutants that may be adsorbed. Recently, CNFs were shown to improve the dispersion of carbon nanotubes [24,25] and graphene [26] in aqueous solutions. Hajian et al. [26] suggested the charges on the TEMPO oxidized CNFs induced an electrostatic stabilization of the CNF–carbon nanomaterial complexes to prevent aggregation. When oxygen-containing carbon nanomaterials, such as graphene oxide (GO) or oxidized CNTs, were used, there was a strong interaction between the oxygen-containing groups of carbon nanomaterials and hydroxyl groups of nanocellulose [27]. Recently, Wei et al. [27] synthesized GO/microcrystalline cellulose (MCC) aerogels in an LiBr aqueous solution. Hybrid GO/MCC aerogel had higher adsorption capacity of MB per unit mass of GO (2630 mg g^−1^) than pure GO when the content of GO was low (0.3 wt%). Hussain et al. [28] fabricated GO/CNF monoliths based on a urea-assisted self-assembly method. The maximum adsorption capacity of these hybrid monoliths to MB achieved 227.27 mg g^−1^. Wu et al. [29] exploited cellulose nanofiber as a cross-linker to interweave between reduced graphene oxide (rGO) layers and obtained hybridized monolith aerogels by a hydrothermal method. The hybridized monolith was able to adsorb not only hydrophilic dyes, but also hydrophobic organic oil. To our knowledge, the influence of carbon nanomaterials features, such as their morphology (nanotubes or nanoplates), on the adsorption behaviors of the CNF/carbon nanomaterial hybrids have not been explored.

Concerning industrial wastewater, different types of dyes could be found. Most of the previous works focus on single solute adsorption in pure water and are not representative of real-world wastewater effluents. During this study, two types of carbon nanomaterials, CNTs and graphene nanoplates (GnPs), are dispersed in water with CNFs using different mass ratios, respectively, to prepare hybrid aerogels through a simple freeze-drying procedure without the assistance of other agents. The aim is to understand the interactions of CNT and GnP with CNF and to explore the adsorption capacity of these hybrid materials to both anionic and cationic dyes in single and binary systems. The preparation, characterization, and adsorption assessments of CNF–CNT and CNF–GnP aerogels for MB and CR dyes are reported. Adsorption behaviors of dyes are inspected by kinetic models and adsorption isothermal models. The adsorption mechanism, contact time, and concentration of MB and CR dye to hybrid aerogels are investigated. Furthermore, desorption of both dyes from the adsorbents are studied.

## 2. Materials and Methods

### 2.1. Materials

Rice straw was harvested at the South China Agricultural University in 2016. Benzene (99.5%, AR, Damao Chemical Reagent Factory, Tianjin, China), ethanol (99.7%, AR, Guangzhou Chemical Reagent Factory, Guangzhou, China), sodium chlorite (NaClO_2_, 80%, Aladdin), acetic acid glacial (CH_3_COOH, 99.5%, AR, Guangzhou Chemical Reagent Factory), potassium hydroxide (KOH, 85%, AR, Shanghai RichJoint Chemical Reagents, Shanghai, China), hydrochloric acid (HCl, 1 N, certified, Guangzhou Chemical Reagent Factory), sodium hydroxide (NaOH, 96%, Guangzhou Chemical Reagent Factory), sodium hypochlorite (NaClO, 8.0%, AR, Damao Chemical Reagent Factory), 2,2,6,6-tetramethylpyperdine-1-oxyl (TEMPO, 98%, Aladdin), sodium bromide (NaBr, 99.9%, AR, Tianjin Fuchen Chemical Reagent Factory, Tianjin, China), methylene blue (MB, AR, Tianjin Fuchen Chemical Reagent Factory), Congo red (CR, Tianjin Fuchen Chemical Reagent Factory), graphene nanoplates (GnPs, Aldrich, grade C-750, thickness: a few nm, particle size: <2 μm, specific surface area: 750 m^2^ g^−1^), and OH-functionalized multi-walled carbon nanotubes (CNTs, Cheap Tubes, external diameter: 50–80 nm, internal diameter: 5–10 nm, length: 10–20 µm, OH content: 5.5%, specific surface area: 60 m^2^ g^−1^ [30]) were used as received. The chemical formula, maximum absorption wavelength and electrical property under neutral pH of MB and CR are shown in Table S1. The deionized (DI) water used was purified by an AIKE Advanced-II-OS water purification system (Cheng Du Kangning Science and Technology Development Company, Chengdu, China).

### 2.2. Preparation of Cellulose Nanofibrils

Pure cellulose was prepared from rice straw by extracting wax and dissolving lignin, hemicellulose and silica [31]. Cellulose nanofibrils (CNFs) were prepared by TEMPO mediated oxidation employing 5 mmol NaClO per gram of cellulose followed by mechanical blending at 37,000 rpm for 30 min [32]. TEMPO-mediated oxidation converted the C6 primary hydroxyls on the surface of the cellulose into carboxyls [33]. The successive mechanical treatment disintegrated oxidized cellulose fibers into individual nanofibrils that were 1–5 nm in width and hundreds of nanometers to 2 μm in length [32]. The carboxylate content of the CNF was measured using a conductometric titration (Oakton CON 6+, Cole-Parmer Instrument Company, Vernon Hills, IL, USA) following a previous method (Gu and Hsieh, 2015) (Figure S1). The carboxylate (COOH + COO^−^) content was determined to be 1.36 mmol/g at neutral pH (COOH content: 0.137 mmol/g).

### 2.3. Preparation of CNF, CNF–CNT and CNF–GnP Aerogels

CNTs or GnPs were added to CNF dispersions with various mass ratios of CNF to carbon nanomaterial (1:0, 3:1, 1:1, 1:3 and 0:1). The final concentration of all the mixtures was controlled to be 6 mg mL^−1^. The mixtures were sonicated by ultrasonic cell disruptor (SCIENTZ, IID, Ningbo, China) at 600 W for 30 min in an ice bath. Each sample was frozen at −21 °C for 4 h in a 15 mL polypropylene centrifuge tube followed by freeze-drying at −50 °C. The CNF–CNT and CNF–GnP hybrid aerogels were obtained. Pure CNF aerogel also was obtained via the same method from a CNF suspension of 6 mg mL^−1^.

### 2.4. Characterization

The dispersion quality of aqueous CNT/CNF and GnP/CNF mixtures after sonication was examined by depositing a drop of each suspension on a glass slide for optical microscope observations. The size of the aggregates was measured and averaged over 150–200 particles. Regarding the compression test, the aerogel samples were transferred into a chamber with a temperature of 25 ± 2 °C and a relative humidity of 65 ± 2%. The compressive strength of the samples was measured 24 h later with a compression ratio of 1 mm min^−1^ on a universal mechanical testing machine (CMT1000, SUST, Zhuhai, China). The morphology of CNF, CNF–CNT, CNF–GnP aerogels and CNT, GnP powder was obtained by a field emission scanning electron microscope (FE-SEM, SU-70, Hitachi, Chiyoda, Japan) after sputtering coating the samples with gold at 15 mA for 2 min under vacuum conditions (Hitachi, E-1010 Ion Sputtering System, Japan). Imaging of the samples was performed at 0.5–30 kV acceleration voltage and 1–2 nA current intensity at magnifications of 20–800,000 times. The chemical structures of CNF, CNF–CNT, CNF–GnP aerogels and CNT, GnP powder were characterized by Fourier transform infrared spectroscopy (FTIR, PerkinElmer, Spectrum 100, Waltham, MA, USA) scanning from 4000 to 400 cm^−1^ with a resolution of 4 cm^−1^. Before FTIR measurements, 2 mg dry sample was ground into powder with 200 mg KBr and pressed into pellets. The specific surface area (SSA) of the samples was calculated using Equation (1) [34].

(1) $$\mathrm{SSA}\;=\;\frac{N_{A}A_{\mathrm{MB}}(C_{o}-C_{e})V}{M_{\mathrm{MB}}M_{S}}$$

where *N*_A_ is Avogadro’s number (6.023 × 10^23^ mol^−1^), *A*_MB_ is the covered area per MB molecules (typically assumed to be 1.35 nm^2^), *C*_o_ and *C*_e_ are the initial and equilibrium concentration of MB, respectively, *V* is the volume of the MB solution, *M*_MB_ is the relative molecular mass of MB, and *M*_S_ is the mass of the sample.

### 2.5. Adsorption of Dyes in Single Systems

Anionic dye CR and cationic dye MB were used in the adsorption experiment. CNF, CNF–CNT, CNF–GnP were aerogels, while pristine CNT and GnP were in the form of powder. Aqueous phase adsorption studies were conducted at 25 °C by submerging 5 mg of a specific nano adsorbent into 20 mL of each dye solution at neutral pH. The mixture was continuously agitated on an orbital shaker at 120 rpm and the amount of residual dye in the solution was determined by ultraviolet-visible spectroscopy (UV-Vis, Thermo Scientific, Evolution 201, Waltham, MA, USA) at the maximum absorption wavelength using a measured extinction coefficient from a Beer’s law analysis for each solution. The adsorption capacity of MB, CR on each adsorbent was calculated using Equation (2).

(2) $$q_{t}=\frac{(C_{0}-C_{t})V}{m}$$

where *q*_t_ is the amount of dye adsorbed at a given time (mg g^−^^1^), *C*_0_ is the initial dye concentration (mg L^−^^1^), *C*_t_ is the residual dye concentration at a given time (mg L^−1^), *V* is the solution volume (L), and *m* is the mass of the adsorbent (mg).

The effect of contact time (0–240 min) was examined using 5 mg of adsorbent and an initial dye concentration of 10, 250 and 500 mg L^−1^ for MB and 100, 600 and 2000 mg L^−1^ for CR. The effect of initial dye concentration on the final adsorption capacity was investigated in a range of dye concentrations (MB: 10, 50, 100, 150, 200, 250, 300, 400, 500, 600, 800 and 1000 mg L^−1^; CR: 10, 50, 100, 150, 200, 250, 300, 400, 500, 600, 800, 1000, 1500 and 2000 mg L^−1^) using an adsorbent of 5 mg at 25 °C and 120 rpm for 16 h.

### 2.6. Adsorption of Dyes in Binary Systems

MB and CR were added into 20 mL DI water with various mass ratios at a total concentration of 200 mg L^−1^. The mass ratio of MB to CR was 3:1, 1:1 or 1:3. An adsorbent of 5 mg was added, and the mixture was agitated at 25 °C and 120 rpm for 16 h. Regarding a binary system, dye A (MB) and dye B (CR) concentrations were calculated as follows:

(3) $$C_{A}=\frac{k_{B2}d_{1}-k_{B1}d_{2}}{k_{A1}k_{B2}-k_{A2}k_{B1}}$$

(4) $$C_{B}=\frac{k_{A1}d_{2}-k_{A2}d_{1}}{k_{A1}k_{B2}-k_{A2}k_{B1}}$$

where *d*_1_ and *d*_2_ are the mixture optical densities measured at *λ*_1_ (664 nm) and *λ*_2_ (498 nm), respectively. *k*_A1_, *k*_B1_, *k*_A2_, and *k*_B2_ are the calibration constants for components A and B at wavelengths of *λ*_1_ and *λ*_2_, respectively [35,36].

### 2.7. Desorption of Dyes

Desorption of MB and CR from CNF, GnP and CNF–GnP 3:1 was performed in ethanol, acetonitrile, acetone or 400 mM NaCl at 25 °C. First, the adsorbents were added into 20 mL of 100 mg L^−1^ dye solutions for adsorption at 120 rpm and then taken out from the dye solutions after 16 h. These adsorbents were transferred to 20 mL of desorption agents. After 1h, the adsorbents were removed and the dye concentration in the solution was measured. The adsorbents might again have been transferred to a fresh desorption agent and the desorption step was repeated for several cycles. Dye percentage removal (%) was calculated by Equation (5):

(5) $$\mathrm{Removal}\;(\%)\;=\;\frac{D_{t}}{C_{0}-C_{e}}\times 100$$

where *D*_t_ is the concentration of dye in the desorption (mg L^−1^), *C*_0_ and *C*_e_ are the initial and final concentrations of dye in the adsorption (mg L^−1^), respectively.

## 3. Results and Discussion

### 3.1. Effect of Adsorbent Structure

**Figure 1:**
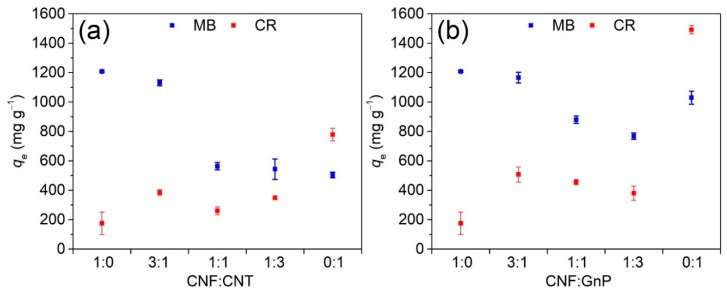
The final adsorption of MB and CR onto CNF–CNT aerogels with the mass ratio of 1:0, 3:1, 1:1, 1:3 and 0:1 (**a**), CNF–GnP aerogels with the mass ratio of 1:0, 3:1, 1:1, 1:3 and 0:1 (**b**). Initial MB concentration was 500 mg L^−^^1^. Initial CR concentration was 2000 mg L^−1^.

Figure 1 shows the uptake of MB or CR after 16 h with constant agitation on different nanosorbents. The initial MB and CR concentrations were 500 mg L^−1^ and 2000 mg L^−1^, respectively. Pure CNF adsorbed the highest amount of cationic MB per unit mass (1207.2 mg g^−1^) due to the large quantity of negatively charged carboxyl groups present on its surface. However, the amount of CR adsorbed on pure CNF was low (175.5 mg g^−1^) possibly due to Coulombic repulsions between negatively charged CNF and anionic CR. Pure GnP powder (1491.7 mg g^−1^) was able to adsorb the highest amount of CR followed by pure CNT powder (777.9 mg g^−1^). The ability of GnP to remove MB was also superior to CNT, which can be attributed to the larger surface area of GnP with more sites available for adsorption. Compared to nanocellulose, carbon nanomaterials could adsorb both cationic and anionic dyes mainly though π–π interaction [37]. Among the hybrid aerogels, CNF–GnP aerogels were generally better than CNF–CNT aerogels in the ability to remove both types of dyes from water at the same CNF to carbon nanomaterial ratio. Moreover, the adsorption capacity of MB onto CNF–GnP 3:1 (1166.1 mg g^−1^) was very close to that of pure CNF. As the quantity of carbon nanomaterials in the hybrid increased, the MB uptake gradually decreased because CNF was displaced by nano adsorbents with lower affinity for MB. Interestingly, among all hybrid aerogels, CNF–GnP 3:1 also exhibited the highest CR uptake (507.1 mg g^−1^), almost two times greater than pure CNF. Similar results were obtained for CNF–CNT hybrids, with CNF–CNT 3:1 having superior MB and CR uptakes than other CNF–CNT combinations. This may suggest that high CNF concentrations can reduce carbon nanomaterial aggregation and improve dispersion quality to promote more effective contact between the solute and the sorbent. *t*-test results indicated the adsorption of MB onto CNF–GnP 3:1 and CNF–CNT 3:1 had no significant difference (*p* > 0.01), but CNF–GnP 3:1 was superior to CNF–CNT 3:1 in the adsorption of CR (*p* < 0.01) (Tables S2–S5). Based on these observations, hybrid aerogels comprising a CNF to carbon nanomaterial ratio of 3:1 were selected for further dispersion, mechanics, morphology, and chemical structure analysis. The CNF–CNT 3:1 and CNF–GnP 3:1 sorbents are reported henceforth as CNF–CNT and CNF–GnP, respectively.

The dispersion states of CNT and GnP aqueous suspensions in the presence of CNFs were investigated by optical microscopy after probe sonication for 5 min and 30 min (Figure S2). After 5 min of ultrasonic treatment, the average particle sizes of CNTs and GnPs were 8.8 ± 13.1 µm (Figure S2a) and 6.6 ± 11.0 µm (Figure S2c), respectively. After 30 min of ultrasonic treatment, large particle aggregations disappeared in each case (Figure S2b,d). The average particle sizes of CNTs and GnPs were 2.1 ± 0.5 µm and 3.1 ± 0.9 µm, respectively. The hydrophobicity of carbon nanomaterials and their tendency to readily form aggregates by hexagonal packing of individual particles with high van der Waals binding energy can greatly reduce the surface area available for adsorption. Ultrasonication provided sufficient energy to break the aggregates of carbon nanomaterials. While TEMPO-oxidized CNFs had negatively charged surface carboxyls, the counterions on the surface of the CNFs induced dipoles in the sp^2^ carbon lattice of the carbon nanomaterials. Then, the charges on the CNFs induced electrostatic stabilization between CNF and CNT/GnP that prevented the carbon nanomaterials from reaggregation [26]. Van der Waals interactions also may occur between CNF and CNT/GnP. CNTs contained few hydroxyl groups and were able to form hydrogen bonds with CNFs. The homogeneous CNF/CNT and CNF/GnP suspensions remained stable for at least 12 h, which was long enough for the preparation of the hybrid aerogels.

**Figure 2:**
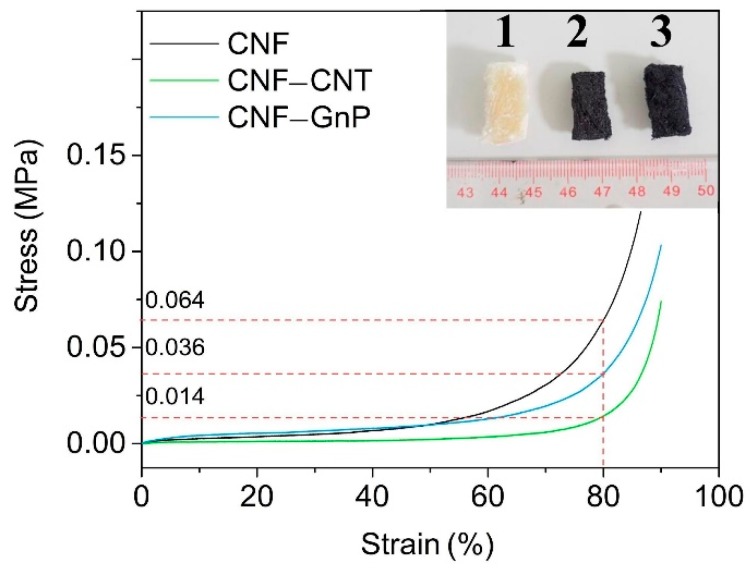
Stress–strain curves of CNF, CNF–CNT and CNF–GnP. The inset is a photograph of the freeze-dried aerogels. 1: CNF, 2: CNF–GnP and 3: CNF–CNT.

Pure CNF aerogel was white with a porous external structure and turned black with the incorporation of carbon nanomaterials (Figure 2, inset). The aerogels formed inside centrifuge tubes during freeze-drying remained intact as cylindrical blocks and could be easily cut into slices using a sharp blade with no apparent deformation. The compression stress-strain curves of the three aerogels are shown in Figure 2. All curves exhibited “slow slope type” before the strain reached 80%. The initial strength of CNF and CNF–GnP aerogels was similar and higher than that of CNF–CNT. After the strains reached 80%, the curves were “steep type”. Inflection points were observed when the strains were approximately 80%. Under the strains of 80%, the compression strengths of CNF, CNF–CNT and CNF–GnP were 0.064, 0.014 and 0.036 MPa, respectively. When immersed in water after compression testing, CNF, CNF–CNT and CNF–GnP aerogels exhibited a water activated shape recovery property (Supplementary Movies S1–S3). The aerogels absorbed water and restored the deformation. Most of the water could be easily squeezed out with tweezers and the compact aerogels could reabsorb water and return to their original size and shape. The fact that the aerogel cylinders were easily squeezed to ~10% of their length and quickly regained the same dimensions with a complete recovery, indicate that the aerogels were mechanically strong, and their open structure allowed the liquid solution to rapidly and freely flow in and out.

**Figure 3:**
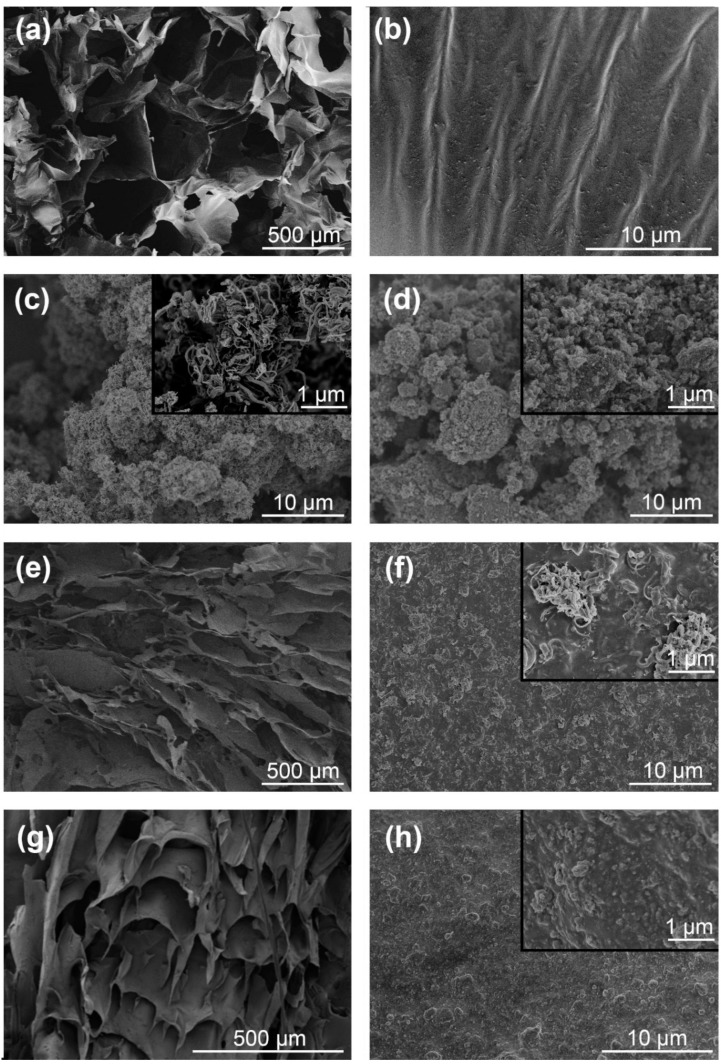
FE–SEM images of CNF aerogel (**a**,**b**), CNT (**c**), GnP (**d**), CNF–CNT aerogel (**e**,**f**) and CNF–GnP aerogel (**g**,**h**). The insets in (**c**,**d**,**f**,**h**) are FE–SEM images of CNT, GnP, CNF–CNT and CNF–GnP at a high magnification, respectively.

The morphologies of the CNF, CNT, GnP, CNF–CNT and CNF–GnP adsorbents have been characterized by SEM (Figure 3a–h). Pure CNF aerogel exhibited a three-dimensional structure with an intercalation of flat and folded sheets and contained pores of various shapes, which may be ascribed to the high suspension concentration (i.e., 6 mg mL^−1^) used before freeze-drying. Pristine CNT and GnP powders revealed the presence of bundles and stacked aggregates due to strong attractive forces between individual particles (Figure 3c,d). The combination between CNT and CNF affected the formation of the CNF sheet structure (Figure 3e). This is the reason that CNF–CNT aerogel produces debris after compression performance testing (Supplementary Movie 2). CNT dispersed better in the CNF matrix (Figure 3f). However, at higher magnification (Figure 3f, inset), some CNT aggregates could be observed still in the matrix. Since both CNFs and CNTs were anisotropic rods, and CNTs were much longer than CNFs, individual CNT partly uncovered by CNFs may re-associate with each other during the freeze-drying process. Thus, the CNT surface active sites available for dye adsorption were reduced. CNF–GnP formed porous aerogels with the main framework still being composed of CNF, while granular GnPs were evenly distributed in the CNFs matrix after ultrasonication and freeze-drying (Figure 3g,h). This can be attributed to the strong interactions between CNF and GnP, preventing GnP stacking and improving the hydrophilicity of GnPs [38]. The graphene platelets were well separated by rod-like CNFs. To contrast with the irregular and aggregated structure of CNT and GnP powders, the hybrid aerogels exhibited an open pore network that can facilitate fast molecular diffusion, hence promoting the accessibility of adsorption sites to relatively large dye molecules. Noteworthy, the morphology of CNF–GnP aerogels was quite different from the curly morphology of pure graphene aerogel in a previous study [39].

**Figure 4:**
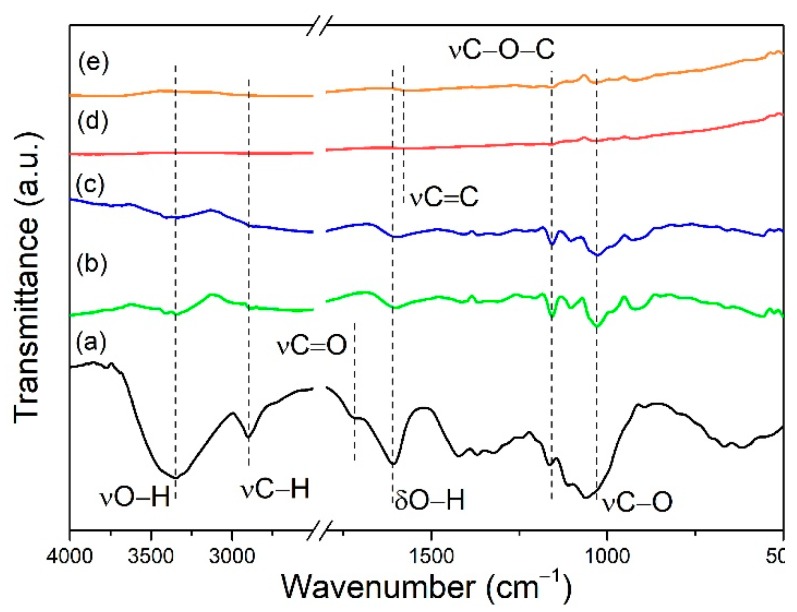
FTIR spectra of CNF (**a**), CNF–CNT (**b**), CNF–GnP (**c**), CNT (**d**) and GnP (**e**). To optimize the representation, the region of 2500–1800 cm^−1^ is omitted.

The FTIR spectra of CNF, CNF–CNT, CNF–GnP, CNT and GnP are shown in Figure 4. The CNF spectrum showed common cellulose peaks: broad hydroxyl stretching at 3360 cm^−1^ and bending at 1610 cm^−1^, predominant C–O peaks at 1168, 1112, and 1062 cm^−1^, and a C–H stretching peak at 2900 cm^−1^, respectively. The small shoulder at 1712 cm^−^^1^ was associated with the carbonyl stretching of the carboxylic acid, confirming C6 primary hydroxyl conversion to carboxyls from TEMPO oxidation [21]. The CNT and GnP spectra were nearly featureless. A small bump at 1570 cm^−1^ was assigned to C=C groups in graphene. Bending vibrations of C–O–C at 1210 cm^−1^ and C–O at 1038 cm^−1^, respectively, indicated epoxide or C–OH structure existing in CNT and graphene. These weak vibration peaks confirmed that the degree of oxidation in CNT and GnP were low. The cellulose characteristic peaks also were observed in the hybrid aerogels containing 25% CNT/GnP. The change of wavenumber for O–H in the hybrids indicated the existence of hydrogen bonding between CNT/GnP and CNF [40]. Since CNT and GnP only had few oxygen containing groups, van der Waals forces and, perhaps, hydrophobic interactions also contributed to the combination of the carbon nanomaterials and CNFs.

Based on adsorption, dispersion, mechanics, morphology and IR results mentioned above, the best performance of the CNF–GnP 3:1 hybrid aerogel perhaps resulted from the plate structure and large surface area of GnPs (i.e., The specific surface areas of GnP and CNT were 750 and 60 m^2^, respectively). A sufficient amount of CNFs prevented GnPs from stacking and improved the hydrophilicity of GnPs. However, CNTs still tangled with each other in the CNF matrix. Dye molecules adsorbed to the GnP portion mainly through π–π and hydrophobic interactions, while cationic MB was able to adsorb to the negatively charged CNF portion by electrostatic interactions. Thus, CNF–GnP 3:1 aerogel was selected for future adsorption kinetics and isothermal modeling.

### 3.2. Effect of Contact Time and Adsorption Kinetics

**Figure 5:**
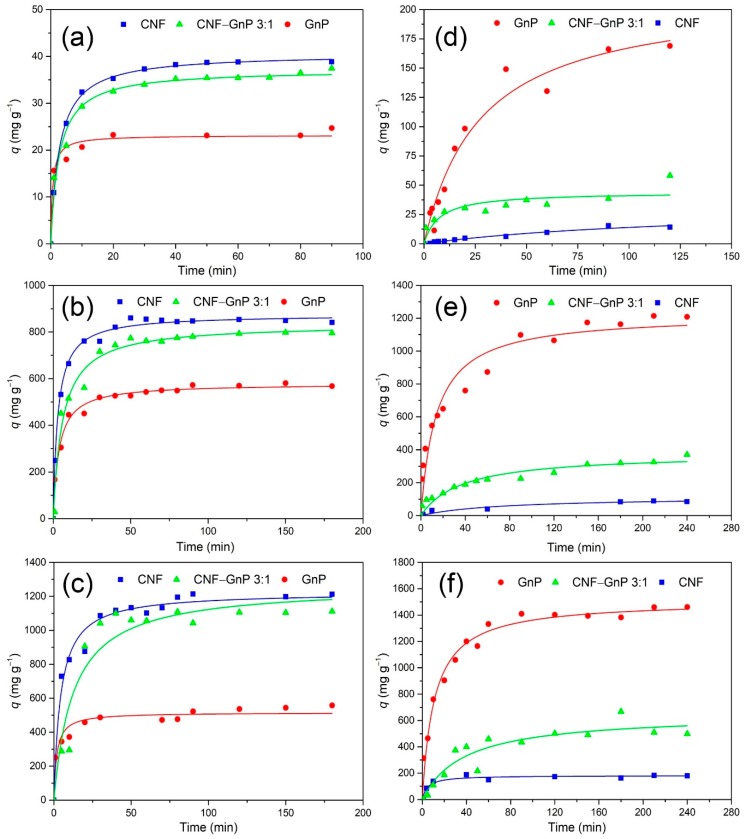
Effects of contact time on the dye removal efficiencies of MB and CR using CNF, CNF–GnP and GnP. Initial MB dye concentration: 10 mg L^−1^ (**a**), 250 mg L^−1^ (**b**), 500 mg L^−1^ (**c**). Initial CR concentration: 100 mg L^−1^ (**d**), 600 mg L^−1^ (**e**), 2000 mg L^−1^ (**f**). Pseudo-second-order adsorption kinetics was applied for all conditions in (**a**–**f**).

The effect of CNF, CNF–GnP and GnP contact time (25 °C, 120 rpm) on dye removal was studied at low, medium and high initial dye concentrations (Figure 5). The initial MB concentrations were 10, 250 and 500 mg L^−1^ and the initial CR concentrations were 10, 600 and 2000 mg L^−1^. The adsorption of MB dye onto CNF, CNF–GnP and GnP occurred rapidly during the first 30 min, then leveled beyond 60 min at all initial MB dye concentrations. The adsorption of CR dye onto CNF, CNF–GnP and GnP occurred at a lower speed compared to that of MB. The adsorption onto CNF and CNF–GnP slowed with adsorption time and reached a plateau beyond 90 min at all initial CR concentrations. However, the adsorption onto GnP still increased very slowly even after 120 min at all initial CR concentrations.

Adsorption kinetics models can be employed to predict the equilibrium adsorption capacity and elucidate the adsorption mechanism. During the adsorption process, the dye molecules migrated from the aqueous solution onto the surface of the adsorbent. MB molecules were adsorbed through electrostatic interactions. The electrostatic interactions occurred when the cationic dye MB was close enough to the adsorption sites (–COO^−^, –OH) on the adsorbent surface. CR molecules were adsorbed mainly through π–π bonding and hydrophobic interactions with carbon nanomaterials. Accompanying the increase in contact time, the accumulation of dye molecules on the adsorbent surface gradually increased and eventually reached equilibrium. The adsorption kinetics of MB and CR on different absorbents was evaluated using both the Lagergren’s pseudo-first-order and the Ho’s pseudo-second-order models. The Lagergren’s pseudo-first-order kinetics is expressed as Equation (6) [41]:

(6) $$q_{t}=q_{e}(1-e^{-k_{1}t})$$

where *k*_1_ is the rate constant (min^−1^), *q*_t_ is the amounts of dye absorbed at a given time (mg g^−1^), and *q*_e_ is the amount of dye adsorbed at equilibrium (mg g^−1^). Nonlinear regression analysis was used to assess the values of *q*_e_, *k*_1_. The Ho’s pseudo-second-order kinetics was expressed as Equation (7) [42]:

(7) $$q_{t}=\frac{q_{e}^{2}k_{2}t}{1+q_{e}k_{2}t}$$

where *k*_2_ is the pseudo-second-order rate constant (g mg^−1^ min^−1^). Nonlinear regression analysis was used to assess the values of *q*_e_, *k*_2_. The initial adsorption rate *v*_0_ at *t* = 0 could be calculated using Equation (8):

(8) $$v_{0}=k_{2}\times q_{e}{}^{2}$$

Regarding all adsorbents, the pseudo-second-order model was generally more applicable for describing the adsorption of MB, as demonstrated by the higher correlation coefficients (*R*^2^), compared to the first-order kinetics (Table 1). This result was consistent with previous reports of cationic dyes adsorbed onto pure CNF [22] and pure graphene [43]. The initial MB adsorption rate (*v*_0_) and MB dye adsorption capacity (*q*_e_) increased rapidly with increasing original dye concentrations from 10 to 250 mg L^−1^ for all adsorbents. Further increasing of the original MB concentration from 250 mg L^−1^ to 500 mg L^−1^ resulted in an increase of *v*_0_ for pure GnP, while *v*_0_ did not change significantly in the cases of pure CNF and CNF–GnP hybrid. This phenomenon may be attributed to the limited amount of negatively charged adsorption sites on the CNF surface at longer contact times and higher MB concentrations. Although the adsorption of MB onto CNF–GnP sorbent was relatively slower than that on each component alone, the hybrid aerogel exhibited the highest theoretical MB adsorption capacity (*q*_e_ = 1264.5 mg g^−1^) at a high initial MB concentration (i.e., 500 mg L^−1^).

The pseudo-second order kinetic model was also clearly a better fit for the adsorption of CR onto pure GnP, which is consistent with a previous study [44]. However, the sorption of CR onto pure CNF could be described as either pseudo first-order kinetics or pseudo second-order kinetics, as indicated by the similar *R*^2^ (0.94–0.98) for both models. Occurring at neutral pH, both CR and CNF were negatively charged. Adsorption of CR onto CNF was relatively low and possibly resulted from hydrophobic interaction. The adsorption of CR onto the CNF–GnP hybrid also could be represented by either the first-order or second-order model and exhibited much higher uptake values than pure CNF. This result possibly indicates that in the hybrid material, CNF did not significantly affect the adsorption kinetics of GnP, even though the GnP content was relatively small. Both the CR adsorption rate (*v*_0_) and CR adsorption capacity (*q*_e_) increased with increasing the initial dye concentrations from 100 to 2000 mg L^−1^ for all adsorbents. The augmentation of the initial concentration provided a greater driving force for the mass transfer and subsequent adsorption on the nanomaterials [45]. The theoretical *q*_e_ value for CNF–GnP reached 648.5 mg g^−1^ at a high initial CR concentration, 2.5 times higher than pristine CNF (182.4 mg g^−1^).

### 3.3. Effect of Initial Dye Concentration and Adsorption Isotherm

**Figure 6:**
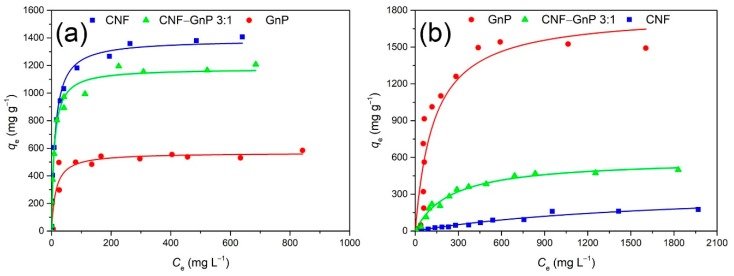
Adsorption of MB (**a**) and CR (**b**) to CNF, CNF–GnP and GnP. The Langmuir adsorption model was applied for data fitting.

The effect of initial dye concentration on the adsorption capacity was investigated in the 10–1000 mg L^−1^ and 10–2000 mg L^−1^ ranges for MB and CR, respectively. The systems were mixed at 25 °C and 120 rpm for 16 h and equilibrium was declared when there was no appreciable change in solution concentration with additional contact time. Concerning all adsorbents, at low dye concentration, adsorption increased dramatically with increasing concentration (Figure 6). The adsorption of MB reached a plateau when residual MB concentration was above 200 mg L^−1^ in all cases. The final MB adsorption at equilibrium for CNF–GnP was 1207.5 mg g^−^^1^ at the initial MB concentration of 1000 mg L^−1^. The adsorption of CR increased relatively slowly when the residual CR concentration was above 400 mg L^−1^ in all cases. The final CR adsorption at equilibrium of CNF–GnP was 507.1 mg g^−1^ at the initial CR concentration of 2000 mg L^−1^.

To further understand the mechanism of adsorption, the adsorbed quantities and residual dyes in the solution at equilibrium were fitted with isothermal models. The adsorption isotherm models describe the interaction between the adsorbate and adsorbent. Three models, Langmuir, Freundlich and Sips, were used to obtain the isotherm parameters for adsorption of dyes onto CNF, GnP, CNF–GnP.

The Langmuir isotherm equation is expressed as follows [46]:

(9) $$q_{e}\;=\;\frac{q_{\max}K_{L}C_{e}}{1+K_{L}C_{e}}$$

where *q*_e_ is the equilibrium adsorption amount per unit weight of the adsorbent (mg g^−^^1^), *C*_e_ is the equilibrium concentration of adsorbate in the solution (mg L^−1^), *q*_max_ is the maximum amount of the dyes adsorbed per unit weight of the adsorbent (mg g^−1^), which describes the complete single-layer coverage on the surface of the dye at a high equilibrium concentration of dyes, and *K*_L_ is the Langmuir adsorption equilibrium constant related to binding site affinity (L g^−1^), representing the bonding energy of adsorbent and adsorption product. The Langmuir isotherm model is based on the monolayer sorption on a surface with a finite number of identical sites and uniform adsorption energies.

The Freundlich isotherm equation is expressed as below [47]:

(10) $$q_{e}=K_{F}\times C_{e}^{\frac{1}{n}}$$

where *K*_F_ (mg g^−1^) and n are the Freundlich constants. The Freundlich isotherm model is an empirical equation for understanding the adsorption of heterogeneous surfaces with multiple adsorption layers. *K*_F_ and n are related to adsorption capacity, adsorption strength and spontaneity, respectively. When the value of *n* is within the range of 1< *n* <10, it indicates a good adsorption process. The larger n value, the better the adsorption effect.

The Sips isotherm equation is given as follows [48]:

(11) $$\frac{1}{q_{e}}=\frac{1}{q_{\max}K_{s}}{\left(\frac{1}{C_{e}}\right)}^{\frac{1}{n}}+\frac{1}{q_{\max}}$$

where *q*_max_ is the Sips constant related to maximum adsorption capacity (mg g^−1^), *K*_S_ is the isotherm constant of Sips related to adsorption energy (L g^−1^), and *n* is the heterogeneity factor. The Sips model is a combination of the Langmuir and Freundlich isotherms. As *K*_S_ approaches 0, the Sips isotherm equation follows the Freundlich model. When *n* approaches or equals 1, the Sips isotherm equation is reduced to the Langmuir isotherm.

The fitting parameters of each model are listed in Table 2. According to the correlation coefficients (*R*^2^), both the Langmuir and Sips adsorption models could adequately describe the dye adsorption on each adsorbent, while the Freundlich isotherm model was the least suitable. Since the Sips model is derived from the Langmuir equation, employs one more fitting parameter, and yields similar correlation coefficients, it could be concluded that the Langmuir model is more appropriate to describe the adsorption behavior. The Langmuir fitting curves for the adsorption of MB and CR on the different adsorbents are shown in Figure 6. The binding constant *K*_L_ is related to the adsorption energy between the adsorbent and the dye. MB displayed higher binding constants (GnP: 7.0 × 10^−2^ L g^−1^, CNF: 8.3 × 10^−2^ L g^−1^, CNF–GnP: 1.1 × 10^−1^ L g^−1^) than CR (GnP: 7.1 × 10^−3^ L g^−1^, CNF: 8.6 × 10^−4^ L g^−1^, CNF–GnP: 3.8 × 10^−3^ L g^−1^) regardless of the adsorbent nature, indicating a higher binding affinity to MB. Compared to the other dye-adsorbent complexes, the uptake of MB on CNF–GnP was the most favorable. The Langmuir model revealed that both MB and CR adsorbed as a monolayer on the CNF–GnP surfaces, with maximum adsorption capacities of 1178.5 mg g^−1^ and 585.3 mg g^−1^, respectively. According to Equation (1) and the monolayer adsorption, the specific surface areas (SSA) of CNF and CNF–GnP were determined to be 3220.4 and 3036.1 m^2^ g^−1^, respectively. Theoretically, if CNF is assumed to be a perfect cylinder with a 1 nm diameter (cellulose density: 1.5 g/cm^3^), the surface area of CNF is 2667 m^2^/g. The high SSA determined by MB adsorption indicates CNFs contained a large number of micropores.

Extensive research about the uptake of various dyes on carbon-based and cellulose/polysaccharide-based composites has been reported in the literature. Table 3 presents a comparison of the maximum dye adsorption capacity of different adsorbents. CNF–GnP was able to adsorb both cationic MB and anionic CR and yielded higher uptake values compared to recent studies using cellulose, activated carbon, graphene, and CNT-based composites.

### 3.4. Adsorption of Dyes in Binary Systems

**Figure 7:**
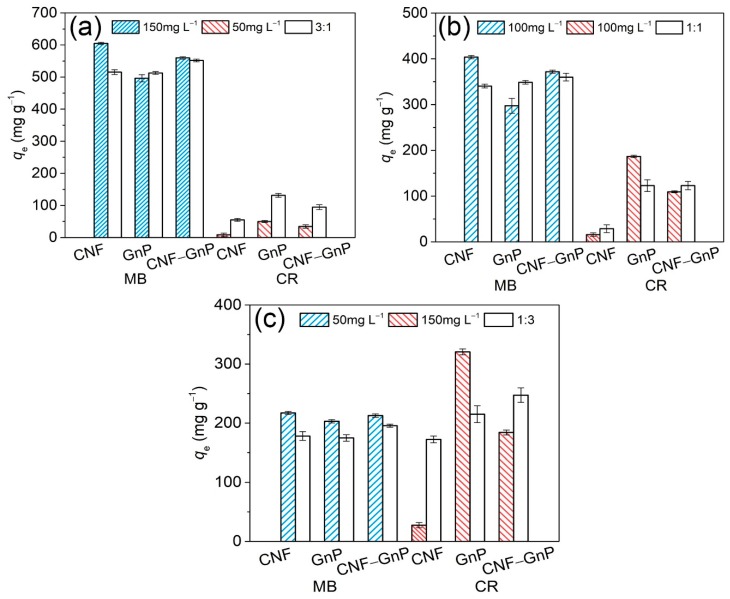
Adsorption of MB and CR in single and binary dye solution. (**a**) mass ratios of MB to CR are 3:1; (**b**) mass ratio of MB to CR are 1:1; (**c**) mass ratio of MB to CR are 1:3. Colored bars represent adsorption in a single dye system. Colorless bars represent adsorption of one dye in a binary dye system.

Regarding industrial wastewater, different types of dyes could be found and they compete for the adsorption sites on the surface of the adsorbent. To investigate the adsorption capacity of CNF–GnP in a more practical setting, an MB and CR binary system was prepared and investigated. The mass ratio of MB to CR in the solution was designed to be 3:1, 1:1 and 1:3. The total initial dye concentration was set to be 200 mg L^−^^1^. The adsorption of dye in single and binary systems was compared (Figure 7). Shown in Figure 7a, when the mass ratio of MB to CR was 3:1 (i.e., MB = 150 mg L^−1^, CR = 50 mg L^−1^), the adsorption of MB onto pure CNF in the binary system was lower than that in the single system, indicating MB and CR competed for adsorption sites on the CNF surface. However, the adsorption of MB onto pure GnP and CNF–GnP was similar in both the single and binary systems. The adsorption of CR onto all adsorbents increased in the binary system compared to that in the single system. This may be attributed to the Coulombic attraction between cationic MB adsorbed on the material surface and anionic CR in solution. Similarly, when the mass ratio of MB to CR was 1:1 (Figure 7b), the adsorption of MB was lower and the CR uptake for pure CNF and CNF–GnP was higher in the binary system than in the single system. However, the adsorption of CR onto pure GnP was strongly affected by the presence of MB when the CR concentration increased. Moreover, when CR became the dominant molecule in the binary system (i.e., MB to CR ratio was 1:3, Figure 7c), the CNF–GnP hybrid was able to adsorb even more CR than pure GnP. Regarding all binary systems, the adsorption capacity of CNF–GnP was the best among the different nano adsorbents, and the presence of one dye had no negative impact on the adsorption of the other dye. The hybrid CNF–GnP adsorbed the highest amount of MB and CR combined per unit mass of adsorbent. Perhaps the cationic MB and anionic CR adsorbed onto different types of adsorption sites on the CNF–GnP surface. The cationic MB mainly adsorbed onto the negatively charged CNF portion, while most of the CR adsorbed onto the GnP portion. MB adsorbed on the material surface also may attract anionic CR in solution via Coulombic attraction.

### 3.5. Desorption

**Figure 8:**
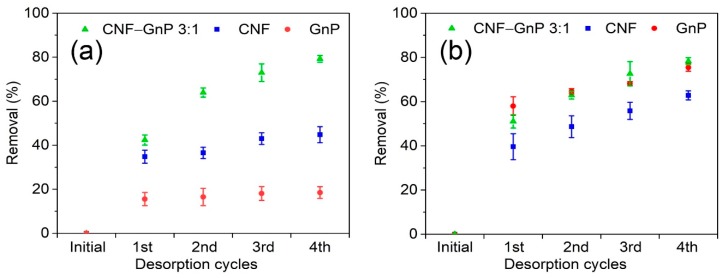
Cyclic desorption of MB (**a**) and CR (**b**) with CNF–GnP, CNF, GnP by ethanol.

The desorption of MB and CR from CNF, GnP and CNF–GnP by ethanol, acetonitrile, acetone and 400 mM NaCl was investigated. The desorption of dye adhered onto CNF, GnP and CNF–GnP was not effective using acetonitrile, acetone and NaCl. Upon immersion in acetonitrile, acetone or 400 mM NaCl for 1 h, only 23.6%, 17.2% or 28.3% of pre-adsorbed MB in CNF–GnP hybrid (100 mg L^−1^ MB, 20 mL MB solution, 16 h) was desorbed, respectively. Upon immersion in acetone or 400 mM NaCl, only 6.0% or 20.1% of pre-adsorbed MB in GnP was desorbed. Concerning CNF, only 36.6% MB was desorbed by acetone. Although 400 mM NaCl could desorb 88.6% MB under the same conditions, the pure CNF aerogel could not remain intact after immersion for 1 h. Strong ionic conditions destroyed the hydrogen bonding network in pure CNF. Dyes adhered to CNF, GnP and CNF–GnP were desorbed rapidly by ethanol (Figure 8). Desorption of MB and CR adhered to CNF–GnP by ethanol was relatively more effective. After 1h of immersion, 42.4% of the MB and 51.0% of the CR were desorbed from CNF–GnP by ethanol. Finally, after four rounds of desorption, 79.2% of the MB and 78.3% of the CR were desorbed. Anhydrous ethanol is a protic solvent that contains polarized oxyhydrogen bonds which ionize to form alkoxyl negative ions and protons (hydrogen ions). It can provide lone pair electron interaction with MB (cationic dye) molecules. Concurrently, anhydrous ethanol also can provide protons with CR (anionic dye) molecules to form hydrogen bonds. The rapid desorption of both MB and CR demonstrate that the CNF–GnP hybrid aerogel could be easily regenerated for repeated dye removal applications.

## 4. Conclusions

Hybrid aerogels containing TEMPO oxidized cellulose nanofibrils (CNFs) and carbon nanomaterials (carbon nanotubes (CNTs) or graphene nanoplates (GnPs)), were designed and synthesized by freeze-drying to remove organic dyes from single and binary systems. When the CNF to GnP mass ratio was 3:1, the hybrid aerogel exhibited the most effective adsorption of both methylene blue (MB) and Congo red (CR) among all the hybrid systems tested. The final adsorption capacities of CNF–GnP 3:1 aerogels for MB and CR reached 1166.1 mg g^−1^ and 507.1 mg g^−1^ at initial dye concentrations of 500 mg L^−1^ and 2000 mg L^−1^, respectively. The CNFs enhanced the dispersion of carbon nanomaterials in an aqueous environment. The hybrid aerogels were mechanically strong and exhibited water-activated shape recovery. Seen in a single dye adsorption system, the adsorption ability measurements demonstrate that the CNF–GnP 3:1 aerogel possessed the adsorption capacity and adsorption rate close to CNF aerogels in cationic MB solutions. The adsorption capacity and adsorption rate of CNF–GnP aerogels was more efficient than the CNF aerogel in an anionic CR solution. Dye adsorptions to CNF–GnP followed a pseudo-second-order adsorption kinetic and the existence of CNF did not affect the adsorption kinetics of GnP. The adsorption followed a monolayer Langmuir isotherm. Concerning a binary system, the CNF–GnP aerogel removed cationic MB as well as anionic CR at a higher total dye adsorption capacity than pristine CNF or GnP. Moreover, 79.2% and 78.3% of the MB and CR were desorbed from CNF–GnP by using ethanol as the desorption agent, suggesting the reusability of this hybrid material. Results of this study indicate that CNF–GnP show promise as high-potential adsorbents for organic dye removal.

## Figures and Tables

**Table 1:** Estimated kinetic parameters of the two adsorption models for methylene blue (MB) and Congo red (CR).

Sample		MB Concentration (mg L^−1^)			CR Concentration (mg L^−1^)		
GnP	Param.	10	250	500	100	600	2000
pseudo-first order	*q*_e_ (mg g^−1^)	22.2	544.2	496.7	167.0	1113.7	1383.5
	*k*_1_ (min^−1^)	1.192	0.170	0.282	0.040	0.049	0.059
	*R^2^*	0.927	0.955	0.856	0.971	0.912	0.960
pseudo-second order	*q*_e_ (mg g^−1^)	23.2	578.3	515.9	213.2	1223.5	1510.4
	*k*_2_ (g mg^−1^ min^−1^)	7.1 × 10^−2^	4.7 × 10^−4^	1.1 × 10^−3^	1.7 × 10^−4^	5.9 × 10^−5^	6.1 × 10^−5^
	*v*_0_ (mg g^−1^ min^−1^)	38.0	157.1	282.2	7.76	87.9	138.4
	*R* ^2^	0.966	0.985	0.937	0.968	0.954	0.981
**CNF**							
pseudo-first order	*q*_e_ (mg g^−1^)	37.8	831.0	1140.0	20.7	92.5	173.4
	*k*_1_ (min^−1^)	0.232	0.198	0.145	0.011	0.012	0.153
	*R^2^*	0.986	0.972	0.933	0.982	0.942	0.960
pseudo-second order	*q*_e_ (mg g^−1^)	40.6	874.8	1225.0	33.0	114.5	182.4
	*k*_2_ (g mg^−1^ min^−1^)	8.9 × 10^−3^	3.8 × 10^−4^	1.9 × 10^−4^	2.2 × 10^−4^	1.20 × 10^−4^	1.2 × 10^−3^
	*v*_0_ (mg g^−1^ min^−1^)	14.7	294.0	278.1	0.241	1.57	38.6
	*R* ^2^	0.999	0.994	0.976	0.978	0.948	0.952
**CNF–GnP 3:1**							
pseudo-first order	*q*_e_ (mg g^−1^)	35.2	767.2	1114.0	37.9	321.4	538.8
	*k*_1_ (min^−1^)	0.210	0.112	0.063	0.128	0.022	0.024
	*R^2^*	0.944	0.961	0.951	0.915	0.931	0.992
pseudo-second order	*q*_e_ (mg g^−1^)	37.2	834.3	1264.5	44.1	379.4	648.5
	*k*_2_ (g mg^−1^ min^−1^)	1.0 × 10^−2^	2.0 × 10^−4^	6.3 × 10^−5^	3.0 × 10^−3^	7.07 × 10^−5^	4.0 × 10^−5^
	*v*_0_ (mg g^−1^ min^−1^)	14.1	137.2	100.2	5.92	10.2	16.9
	*R* ^2^	0.978	0.977	0.910	0.940	0.955	0.972

**Table 2:** Estimated adsorption parameters of Langmuir, Freundlich and Sips isotherms at room temperature.

Langmuir Adsorption Model		GnP		CNF		CNF–GnP 3:1	
		MB	CR	MB	CR	MB	CR
*q*_e_ = (*q*_max_*K*_L_*C*_e_)/(1 + *K*_L_*C*_e_)	*q*_max_ (mg g^−1^)	567.1	1787.3	1387.2	351.7	1178.5	585.3
	*K*_L_ (L g^−1^)	7.0 × 10^−2^	7.1 × 10^−3^	8.3 × 10^−2^	8.6 × 10^−4^	1.1 × 10^−1^	3.8 × 10^−3^
	*R* ^2^	0.879	0.858	0.984	0.960	0.985	0.980
**Freundlich adsorption model**		**GnP**		**CNF**		**CNF** **–GnP 3:1**	
		**MB**	**CR**	**MB**	**CR**	**MB**	**CR**
*q*_e_ = *K*_F_ × *C*_e_^1/*n*^	*n*	5.431	2.995	4.512	1.428	4.984	1.944
	*K*_F_ (mg g^−1^)	181.3	156.1	376.5	0.966	364.2	29.9
	*R* ^2^	0.773	0.719	0.902	0.935	0.892	0.864
**Sips adsorption model**		**GnP**		**CNF**		**CNF** **–GnP 3:1**	
		**MB**	**CR**	**MB**	**CR**	**MB**	**CR**
1/*q*_e_ = (1*/q*_max_*K*_s_) × (1/*C*_e_)^1/*n*^ + (1/*q*_max_)	*q*_max_ (mg g^−1^)	552.4	1515.0	1453.0	235.2	1231.1	517.9
	*K*_s_ (L g^−1^)	4.1 × 10^−2^	2.3 × 10^−4^	1.2 × 10^−1^	1.0 × 10^−4^	1.5 × 10^−1^	6.8 × 10^−4^
	*n*	0.819	0.534	1.222	0.727	1.243	0.723
	*R* ^2^	0.869	0.885	0.991	0.960	0.988	0.969

**Table 3:** Comparison of adsorption capacity of different adsorbents.

Adsorbent	*q*_max_ (mg g^−1^) MB	Ref.	Adsorbent	*q*_max_ (mg g^−1^) CR	Ref.
CNF–GnP aerogel	1178.5	this study	CNF–GnP aerogel	585.3	this study
Zeolite—activated carbon composite from oil palm ash	285.71	[49]	Polyaniline@GO-multiwalled carbon nanotube nanocomposite	66.67	[44]
Nickel nanoparticles/porous carbon—carbon nanotube hybrids	312	[50]	Chitosan hydrogel beads impregnated with CNT	450.4	[51]
3D rGO/L–Cys hydrogel	660	[52]	Functionalized multiwalled carbon nanotubes	148	[53]
Graphene/cellulose nanofibers	227.27	[28]	Polyacrylamide grafted quaternized cellulose	349.28	[54]
GO/calcium alginate composites	181.81	[55]	CaCO_3_−cellulose aerogel	75.81	[56]

## Supplementary Materials

The following are available online at https://www.mdpi.com/2079-4991/10/1/169/s1, Figure S1: Conductometric titration curves of CNF (a) with HCl added and (b) without HCl added., Figure S2: Optical micrographs of (a) CNF/CNT 3:1 after 5 min ultrasonic treatment; (b) CNF/CNT 3:1 after 30 min ultrasonic treatment; and (c) CNF/GnP 3:1 after 5 min of ultrasonic treatment; (d) CNF/GnP 3:1 after 30 min ultrasonic treatment, Table S1: Chemical structure, molecular weight, maximum absorption wavelength and electrical property of methylene blue and Congo red. Table S2: Group statistics of the final adsorption of MB. Table S3: Independent sample tests of the final adsorption of MB onto CNF–GnP 3:1 and CNF–CNT 3:1. Table S4: Group statistics of the final adsorption of CR. Table S5: Independent sample tests of the final adsorption of CR onto CNF–GnP 3:1 and CNF–CNT 3:1. Movie S1: Shape recovery of CNF aerogel in water after compression. Movie S2: Shape recovery of CNF–CNT aerogel in water after compression. Movie S3: Shape recovery of CNF–GnP aerogel in water after compression in water.
