# Supplementary material for: Cellulose Nanofibril/Carbon Nanomaterial Hybrid Aerogels for Adsorption Removal of Cationic and Anionic Organic Dyes
Source: Nanomaterials (Basel). 2020 Jan 19;10(1):169. doi: 10.3390/nano10010169 (PMC7022903; doi:10.3390/nano10010169)
Supplement: Supplementary file 1 [file nanomaterials-10-00169-s001.zip › nanomaterials-669824-SP/nanomaterials-669824-SP.pdf]

Supplementary Material

Cellulose Nanofibril/Carbon Nanomaterial Hybrid Aerogels for Adsorption Removal of Cationic and Anionic Organic Dyes

Zhencheng Yu [1], Chuanshuang Hu [1,*], Anthony B. Dichiara [2], Weihui Jiang [1] and Jin Gu [1,*]

[1] College of Materials and Energy, South China Agricultural University, Guangzhou 510642, China; yuzansing2017@stu.scau.edu.cn (Z.Y.); 201820117385@mail.scut.edu.cn (W.J.)

[2] School of Environmental and Forest Sciences, University of Washington, Seattle, WA 98195, USA; abdichia@uw.edu

* Correspondence: cshu@scau.edu.cn (C.H.); gujin@scau.edu.cn (J.G.); Tel.: +86-20-85282568 (C.H.); +86-20-85280319 (J.G.)

Figures

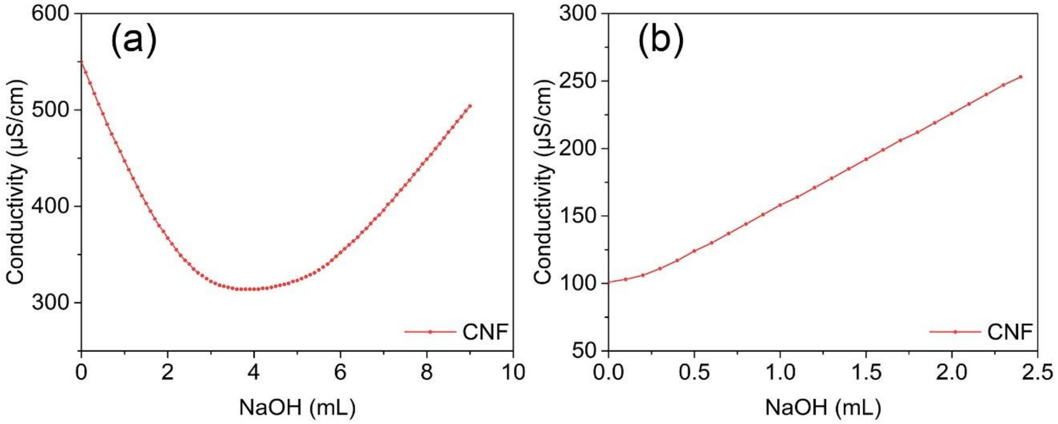

Figure S1 Conductometric titration curves of CNF (a) with HCl added and (b) without HCl added. In Figure a, before titration, hydrochloric acid of 1 mL (0.1N) was added to CNF suspension (0.1 wt%) of 50 mL to protonate the carboxyl groups. After that, the suspension was titrated with 0.02 M NaOH solution. The COOH+COO- content of the original CNF was calculated based on the NaOH amount added in the plateau region. In Figure b, the CNF suspension (0.1 wt%) of 50 mL was directly titrated with 0.02 M NaOH solution. The COOH content of the original CNF was calculated based on the NaOH amount added in the plateau region.

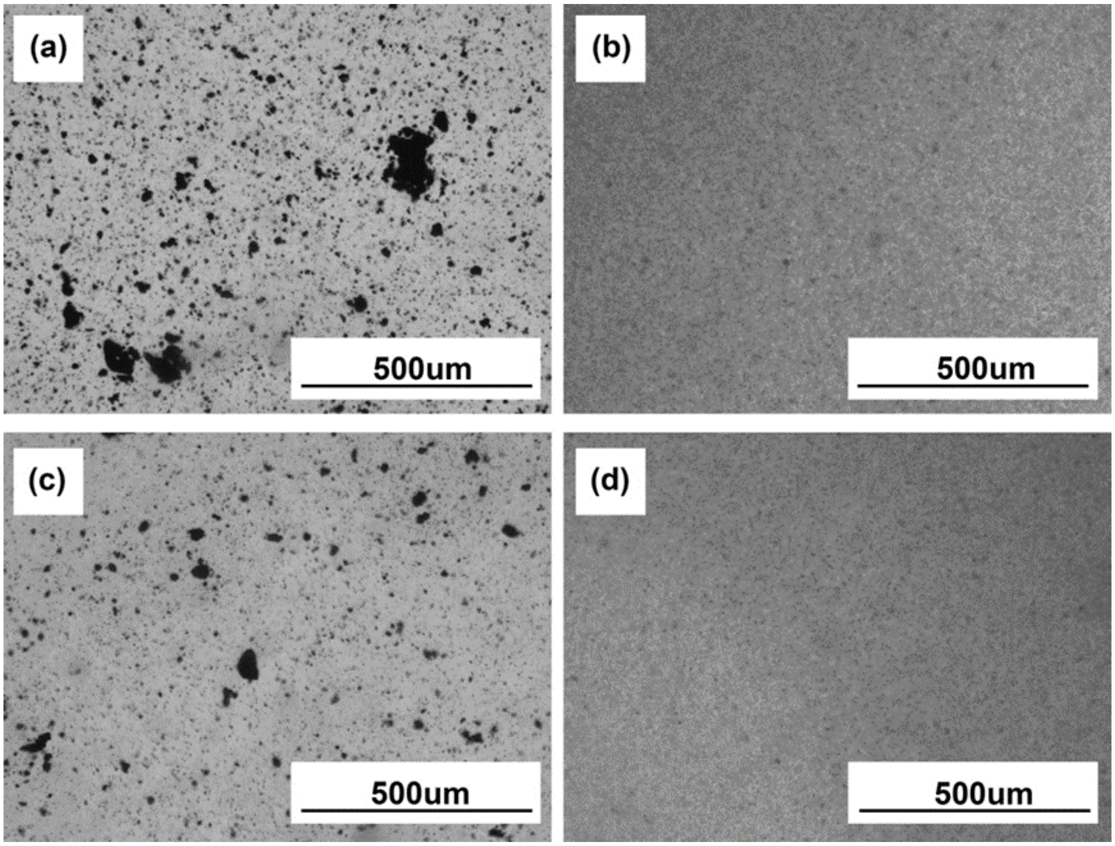

Figure S2. Optical micrographs of (a) CNF/CNT 3:1 after 5 min ultrasonic treatment; (b) CNF/CNT 3:1 after 30 min ultrasonic treatment; and (c) CNF/GnP 3:1 after 5 min ultrasonic treatment; (d) CNF/GnP 3:1 after 30 min ultrasonic treatment.

Tables

Table S1. Chemical structure, molecular weight, maximum absorption wavelength and electrical property of methylene blue and Congo red.

Dyes	Chemical formula	Molar mass ($g\ mol^{-1}$)	λ_{max}(nm)	Electrical property
methylene blue (MB)	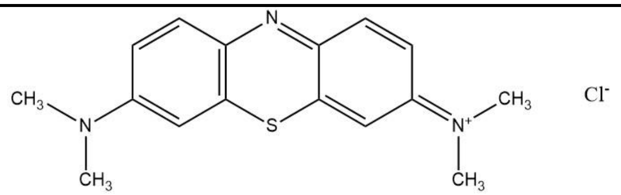	319.9	664	Cationic
Congo red (CR)	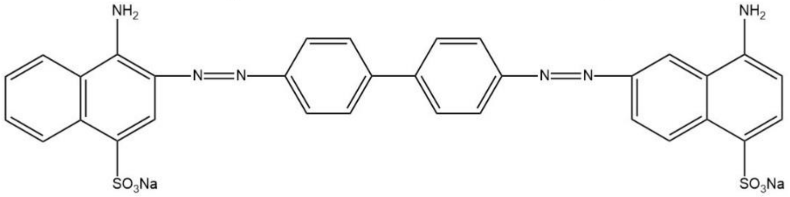	696.7	498	Anionic

Table S2. Group statistics of the final adsorption of MB.

	Type	N	Mean	Std. Deviation	Std. Error Mean
The final adsorption of MB	CNF–GnP 3:1	3	1166.082	25.7257	14.8528
	CNF–CNT 3:1	3	1130.145	14.2890	8.2498

Table S3. Independent samples test of the final adsorption of MB onto CNF–GnP 3:1 and CNF–CNT 3:1. Whether these two materials had significantly different effects on the final adsorption of MB was investigated. Sig.=0.48>0.05, so the variances are equal. In this case, Sig.(2-tailed) =0.102>0.01. Therefore, the effect of these two materials on the final adsorption of MB had no significant difference.

		Levene's Test for Equality of Variances		*t-test for Equality of Means*						
		F	Sig.	t	df	Sig.(2-tailed)	Mean Difference	Std. Error Difference	95% Confidence Interval of the Difference	
									Lower	Upper
The final adsorption of MB	Equal variances assumed	.604	.480	2.115	4	.102	35.9370	16.9901	-11.2350	83.1090
	Equal variances not assumed			2.115	3.127	.121	35.9370	16.9901	-16.9136	88.7875

Table S4. Group statistics of the final adsorption of CR

	Type	N	Mean	Std.Deviation	Std.Error Mean
The final adsorption of CR	CNF–GnP 3:1	3	507.059	36.0784	20.8299
	CNF–CNT 3:1	3	381.387	14.1596	8.1751

Table S5. Independent samples test of the final adsorption of CNF–GnP 3:1 and CNF–CNT 3:1to CR. Whether these two materials had significantly different effects on the final adsorption of CR was investigated. Sig. = 0.336 > 0.05, so the variances are equal. In this case, Sig.(2-tailed) = 0.005 < 0.01. Therefore, the effect of these two materials on the final adsorption of CR had significant difference.

		Levene's Test for Equality of Variances		*t-test for Equality of Means*						
		F	Sig.	t	df	Sig.(2-tailed)	Mean Difference	Std. Error Difference	95% Confidence Interval of the Difference Lower	Upper
The final adsorption of MB	Equal variances assumed	1.191	.336	5.616	4	.005	125.6722	22.3767	63.5445	187.7998
	Equal variances not assumed			5.616	2.602	.016	125.6722	22.3767	47.8795	203.4648
